# Pyroptosis in periodontitis: From the intricate interaction with apoptosis, NETosis, and necroptosis to the therapeutic prospects

**DOI:** 10.3389/fcimb.2022.953277

**Published:** 2022-08-16

**Authors:** Xiaohui Xu, Tingwei Zhang, Xuyun Xia, Yuanyuan Yin, Sihan Yang, Dongqing Ai, Han Qin, Mengjiao Zhou, Jinlin Song

**Affiliations:** ^1^ College of Stomatology, Chongqing Medical University, Chongqing, China; ^2^ Chongqing Key Laboratory of Oral Diseases and Biomedical Sciences, Chongqing, China; ^3^ Chongqing Municipal Key Laboratory of Oral Biomedical Engineering of Higher Education, Chongqing, China; ^4^ Department of Endocrinology, The Second Affiliated Hospital, Chongqing Medical University, Chongqing, China

**Keywords:** pyroptosis, periodontitis, programmed cell death, diabetes, cardiovascular diseases, rheumatoid arthritis

## Abstract

Periodontitis is highly prevalent worldwide. It is characterized by periodontal attachment and alveolar bone destruction, which not only leads to tooth loss but also results in the exacerbation of systematic diseases. As such, periodontitis has a significant negative impact on the daily lives of patients. Detailed exploration of the molecular mechanisms underlying the physiopathology of periodontitis may contribute to the development of new therapeutic strategies for periodontitis and the associated systematic diseases. Pyroptosis, as one of the inflammatory programmed cell death pathways, is implicated in the pathogenesis of periodontitis. Progress in the field of pyroptosis has greatly enhanced our understanding of its role in inflammatory diseases. This review first summarizes the mechanisms underlying the activation of pyroptosis in periodontitis and the pathological role of pyroptosis in the progression of periodontitis. Then, the crosstalk between pyroptosis with apoptosis, necroptosis, and NETosis in periodontitis is discussed. Moreover, pyroptosis, as a novel link that connects periodontitis with systemic disease, is also reviewed. Finally, the current challenges associated with pyroptosis as a potential therapeutic target for periodontitis are highlighted.

## Introduction

Pyroptosis has been demonstrated to participate in the pathophysiological processes of various inflammatory diseases, such as coronavirus disease—2019, colitis, rheumatoid arthritis, Crohn’s disease, and neuroinflammatory injury (Xu et al., 2019; Wu et al., 2020; Cai et al., 2021; Tan et al., 2021; Vora et al., 2021). Pyroptosis is categorized as inflammatory programmed cell death that is mainly dependent on caspases and gasdermins (GSDMs). The canonical pyroptosis pathway is initiated by the activation of the NOD-like receptor (NLR) family pyrin domain containing 3 (NLRP3), followed by caspase-1-mediated cleavage and activation of GSDMD, pro-interleukin (IL)-1β, and pro-IL-18. Mature GSDMD (N-terminal fragment of GSDMD) ultimately assembles on the plasma membrane and forms pore-like structures, allowing water to enter cells and enabling the excretion of cellular contents and mature inflammatory factors (Yu et al., 2021). This determines the distinct morphological characteristics of pyroptosis compared to other forms of programmed cell death. Recent studies on pyroptosis have reported that apoptosis-associated caspase-3 and 8 can also mediate the cleavage of GSDMs, thus mediating the activation of pyroptosis (Sarhan et al., 2018; Jiang et al., 2020). This highlights the crosstalk between pyroptosis and apoptosis. According to the existent proofs of pyroptosis in infectious diseases, appropriate pyroptosis activation is beneficial in defending and clearing foreign pathogens, while aberrant activation can cause uncontrolled inflammatory responses and tissue damage. Hence, there are two sides to pyroptosis in the pathophysiological processes of periodontitis, with the potential to be either beneficial or detrimental to the host’s defense and periodontal tissue regeneration.

Periodontitis is initiated by infection with pathogenic microorganisms; inadequate treatment results in long-term plaque accumulation, leading to a sustained inflammatory response and irreversible loss of periodontal tissues. Genetics, treatments, and self-performed oral hygiene all affect the prognosis of periodontitis (Kinane et al., 2017). Among the reported mechanisms, various programmed cell death pathways have been reported to be involved in the progression of periodontitis. To date, more than ten kinds of programmed cell death pathways have been identified, and studies have addressed their roles in the pathogenesis of periodontitis. While apoptosis is well studied in periodontitis, there is still a lack of an in-depth investigation of pyroptosis and its relation to apoptosis, necroptosis, and NETosis in this field. Pyroptosis was reported to be associated with an extensive inflammatory reaction in periodontitis. Clinical studies of apical periodontitis (AP) have shown that the level of periodontal pyroptosis correlates positively with disease severity (Cheng et al., 2018). Furthermore, an increased level of pyroptosis in periodontal tissue leads to overactive immune responses and promotes the secretion of active inflammatory factors (IL-1β, IL-18), further contributing to the overactivation of inflammatory signaling pathways. This, in turn, leads to reduced bone formation ability via the suppression of osteoblast activity and enhanced bone resorption via the upregulation of the proliferation and activity of osteoclasts (Li et al., 2021). Ultimately, this results in the exacerbation of the destruction of periodontal tissue and the suppression of its regeneration (Li et al., 2021). The exact mechanisms will be discussed below.

Recent studies have broadened our understanding of the role of pyroptosis in periodontitis. However, there remains a significant gap in comprehending the specific molecular mechanisms of pyroptosis in periodontitis, and the favorable aspect of pyroptosis in periodontitis, regarding its role in clearing pathogens, also requires further verification. This review summarizes the most recent contributions to our understanding of the potential mechanisms underlying the role of pyroptosis in the pathogenesis and development of periodontitis. Moreover, the crosstalk between pyroptosis and apoptosis, necroptosis, and NETosis is discussed. Based on this review, it is hypothesized that pyroptosis might be a novel target that connects periodontitis with systemic disease. Finally, the recent progress and challenges in the translation of pyroptosis research into therapeutic targets are summarized to reveal the new therapeutic options applicable to periodontitis.

## Mechanisms of pyroptosis activation in periodontitis

Pyroptosis is initiated by canonical and noncanonical activation of inflammasomes. In the canonical pathway, pathogen-associated molecular patterns (PAMPs) or damage-associated molecular patterns (DAMPs) bind to the major histocompatibility complex or pattern recognition receptors (PRRs), which leads to the increased transcription and translation of inflammasome constituents, and also promotes the activation of inflammasomes by oligomerization and recruitment of the components of the inflammasomes (Rathinam and Fitzgerald, 2016). Inflammasomes are heterologous oligomeric protein complexes usually comprised of NLRs or absent in melanoma 2 (AIM2)-like receptors (ALRs), apoptosis-associated speck-like proteins containing a caspase recruitment domain (ASC), and pro-caspase-1 (Li et al., 2021). Among the 23 identified NLRs in humans, only a few of them participate in the formation of inflammasomes; they include NLRP3, NLRP1, NLRP6, and NLRC4. NLRs usually possess a leucine-rich repeat (LRR) domain at the C-terminal, a nucleotide-binding domain (NBD) or a nucleotide-binding and oligomerization (NACHT) domain in the central region, and a pyrin domain (PYD) at the N terminal of the NLRP or a caspase recruitment domain (CARD) at the N terminal of the NLRC (Swanson et al., 2019; Li et al., 2021; Yu et al., 2021). Following the activation of inflammasomes, the PYD in the NLRP, such as NLRP3, assembles pro-caspase-1 (full-length) by indirectly binding to the ASC (containing a CARD at the C-terminal and a PYD at the N-terminal) of the inflammasome via the homotypic interaction between PYD–PYD and CARD–CARD. However, the CARD in NLRP1 and NLRC4 can promote the recruitment of pro-caspase-1 by directly binding to their CARD domains (Kay et al., 2020; Taabazuing et al., 2020; Sundaram and Kanneganti, 2021). Once bound to inflammasomes, there is the promotion of the dimerization and autoproteolysis of pro-caspase-1, followed by the release of central and small catalytic domains, p20 and p10 (Guo et al., 2015; Huang et al., 2021; Li et al., 2021).

Unlike in the canonical pathway, in the noncanonical pathway, caspase-4/5 in humans and caspase-11 in mice function as both sensor and effector molecules of lipopolysaccharide (LPS)-induced pyroptosis. After the recognition of LPS, caspase-4/5/11 dimerization and autoproteolysis occur, releasing the p10 and p20 domains (Kayagaki et al., 2015; Downs et al., 2020). Recent advances in this field have indicated that several guanylate binding proteins (GBPs) are involved in caspase-4 signaling induced by LPS (Fisch et al., 2020; Kutsch et al., 2020; Santos et al., 2020; Wandel et al., 2020); however, this requires further investigation in periodontitis. The active caspases in the canonical and noncanonical pathways ultimately proteolytically cleave pro-IL-1β and pro-IL-18 into their biologically active forms. Although caspase-4/5/11 cannot directly cleave pro-IL-1β and pro-IL-18, they can promote the maturation and secretion of IL-1β and IL-18 by activating the NLRP3/caspase-1 pathway (Yu et al., 2021). Simultaneously, the active caspase-1/4/5/11 cleaves the GSDMD protein, the key effector protein of pyroptosis, to form active N- and C-terminal kinase portions. The N-terminal of GSDMD binds to phosphatidylserine, phosphatidic acid, and phosphatidylinositol on the cell membrane, promoting oligomerization and resulting in the formation of pore-like structures on the cells. Changes in membrane permeability lead to cell swelling and membrane rupture, which facilitate the active forms of inflammatory factors to pass through the membrane pores. Moreover, new evidence indicates that the GSDMD pore conduit is predominantly negatively charged, thus favoring the passage of positively charged and neutral cargo, such as the mature forms of IL-1β and IL-18 (Xia et al., 2021).

As mentioned above, GSDMD is the most studied member of the GSDM family, and it mediates both the canonical and noncanonical pathways. GSDMA, -B, -C, and -E, as well as autosomal recessive deafness-59 (DFNB59), are less studied and require further investigation. Aside from DFNB59, all GSDMs have a pore-forming domain at the N-terminal, an autoinhibitory domain at the C-terminal, and a loop domain that links the N- and C-terminal domains (Julien and Wells, 2017; Kovacs and Miao, 2017; Xia et al., 2020). Except for DFNB59, they were proved to be involved in the activation of pyroptosis. Restricted reports showed that GSDME can be cleaved by caspase-3, which specifically generates an N-terminal fragment of GSDME, thus promoting pyroptosis (Wang et al., 2017; Jiang et al., 2020). Moreover, GSDMC was reported to be cleaved by caspase-8, specifically with TNFα treatment, generating an N-terminal fragment of GSDMC, thereby inducing pyroptosis (Hou et al., 2020; Zhang et al., 2021). Caspase-8 has also been reported to cleave GSDMD to induce pyroptosis (Orning et al., 2018; Sarhan et al., 2018; Demarco et al., 2020). These results also indicated that GSDME and GSDMC might be two points linking pyroptosis with apoptosis. This will be discussed below. Aside from caspases, granzyme A has been reported to cleave GSDMB in lymphocytes (Zhou et al., 2020), and granzyme B was reported to directly cleave GSDME in tumor cells (Zhang et al., 2020), ultimately contributing to tumor suppression by invoking pyroptosis. In addition, a recent study reported that neutrophil-specific serine protease-neutrophil elastase (ELANE) can also cleave GSDMD at the N terminal to promote pyroptosis, thereby mediating its biological effects (Kambara et al., 2018). However, non-caspases-dependent cleavage of GSMDs was not clarified in periodontitis. Therefore, detailed molecular and biological investigations need to be conducted to shed light on it.

Accumulating evidence has shown that pyroptosis participates in the pathophysiological process of periodontitis (Bullon et al., 2018; Zhou et al., 2020; Li et al., 2021). Continuous pathogenic microorganism infection is the initiating factor in periodontitis, including bacterial, fungal, viral, and mycoplasma infections. Toll-like receptor 4 (TLR4) is the most characterized pattern recognition receptor during periodontitis and has been largely argued for its crucial role in the LPS-mediated pyroptosis pathway. Basic studies using TLR4 knockout mice models have documented that TLR4 is involved in periodontitis and peri-implantitis initiated by P. gingivalis (Lin et al., 2014; Deng et al., 2020). Recent advances documented that after recognizing and binding to the lipid A portion of LPS by TLR4 particularly, the myeloid differetial protien-2 mediates the binding of LPS with TLR4, followed by initiating the homotypic interaction of TLR4’s intracellular toll/interleukin-1 receptor domain with adaptors, including myeloid differentiation factor88 (MyD88) and TIR domain-containing adapter protein inducing IFN-Beta (TRIF). Consecutively, MyD88 binds to interleukin-1 receptor-associated kinase (IRAK) 1 and 2, which facilitates the assembly of TRAF6 and provokes TAK1-mediated phosphorylation and activation of IκB kinases α/β (IKKα/β) (Ciesielska et al., 2021), ultimately leading to nuclear translocation of NF-kB. This step is essential for the transcription of NLRP3, pro-IL-1β, and pro-IL-18, the priming step for the activation of NLRP3 inflammasome (Ciesielska et al., 2021). In the TRIF-dependent pathway, TRAF3 and TRAF6 were involved in the activation of the MAPK and ERK1/2 pathways, which also contributed to cytokine production (Ciesielska et al., 2021). The activation of NLRP3 was demonstrated to be associated with posttranslational modification, which was supported by NLRP3 deubiquitination by BRCC3 or phosphorylation by JNK1, promoting the activation of the NLRP3 inflammasome (Py et al., 2013; Song et al., 2017). In addition, ion flux, mitochondrial damage, ROS and mitochondrial DNA, and lysosomal disruption were also documented to be involved in NLRP3 activation (Huang et al., 2021). However, the exact underlying mechanisms are still obscure. In addition to recruiting and activating inflammasomes in the canonical pyroptosis pathway, downstream molecular TRIF initiates the activation of IRF3/7 and the induction of type I interferon release in parallel. This is followed by IFNAR1/2-dependent activation of JAK/STAT signaling to initiate pro-caspase-11 expression, thus participating in the noncanonical pyroptosis pathway (Gurung et al., 2012; Rathinam et al., 2012; Zamyatina and Heine, 2020). These findings extend our understanding of LPS and TLR4 in pyroptosis. However, these results have not been verified in periodontitis. Pyroptosis was demonstrated to arise in human gingival fibroblasts (HGFs), macrophages, oral epithelial cells, human periodontal ligament fibroblasts (HPDLFs), human periodontal ligament stem cells (HPDLSCs), and osteoblasts during periodontitis (Ziauddin et al., 2018; Zhuang et al., 2019; Chen et al., 2021; Lei et al., 2021; Li et al., 2021; Zhao et al., 2021). The human gingival epithelium (HGEs) is considered the first line of periodontal defense, protecting the periodontal tissue against various harmful pathogens. Streptococcus sanguinis and oral anaerobic bacteria-produced butyrate can destroy the epithelial barrier through the activation of pyroptosis by upregulating caspase-3/GSDME and caspase-1, respectively (Liu et al., 2019; White et al., 2020). HGFs are the most abundant cells in the periodontal tissue; activation of the pyroptosis pathway in gingival fibroblasts contributes to the exacerbation of inflammation and the destruction of periodontal tissues. Porphyromonas gingivalis (P. gingivalis) and LPS are reported to induce pyroptosis in HGFs by activating the NLRP3/NLRP6/caspase-1/GSDMD pathway (Liu et al., 2018; Huang et al., 2020; Yang et al., 2021). Moreover, caspase-4/GSDMD, which belongs to the noncanonical pathway, can also be activated in HGFs in response to the Treponema pallidum surface protein Tp92 (Jun et al., 2018). Macrophages are key mediators of the inflammatory response in the innate immune system and are involved in defense against pathogen invasion by recognizing the byproducts of pathogens and other endogenous factors. Nonetheless, overactivation of microphages results in an extended inflammatory response and tissue damage (Shapouri-Moghaddam et al., 2018). In addition, macrophages are remarkable plastic cells that can be phenotypically polarized to classically activated or inflammatory (M1) and alternatively activated or anti-inflammatory (M2) forms. While M2 can be induced by IL-4 and IL-13, which is followed by the production of anti-inflammatory factors IL-10 and TGF-β, M1 is triggered by recognizing IFN-γ, TNF-α, or LPS with TLRs and IL-1R, and this is followed by the production of pro-inflammatory cytokines TNF-α, IL-1α, IL-1β, IL-6, IL-12, and IL-23 (Murray, 2017; Locati et al., 2020). Cytokine production during this process was activated NF-κB signaling dependent (Murray, 2017; Locati et al., 2020). These results showed that M1 polarization shares the same initiated signaling pathways with pyroptosis. Concomitant with these findings, M1 macrophages showed increased caspase-1 expression (Xia et al., 2022), and polarization to M1 can be prevented by caspase-1 suppression (Li et al., 2019). So, we speculate that in response to infection, M1 polarization occurs in the early stage, while accumulating inflammatory factors produced by M1 contribute to pyroptosis activation. Given that M2 macrophages have scavenger receptors, they might participate in the phagocytosis of cells undergoing pyroptosis, thus restricting the amplification of inflammation. However, the interaction between macrophage polarization and pyroptosis has never been investigated in periodontitis; gene knockout of pyroptosis-related caspases will help us understand this topic. Limited evidence in periodontitis revealed that P. gingivalis, Mycoplasma salivarium, Treponema denticola surface protein Td92, E. faecalis, and LPS can induce pyroptosis in macrophages through the NLRP3/caspase-1/GSDMD pathway, contributing to the pathogenesis and development of periodontitis (Jun et al., 2012; Sugiyama et al., 2016; Li et al., 2019; Li et al., 2020; Ran et al., 2021). Moreover, pyroptosis induced by the cyclic stretch, dental calculus, and outer membrane vesicles of P. gingivalis in macrophages can also promote periodontitis (Fleetwood et al., 2017; Ziauddin et al., 2018; Zhuang et al., 2019).

**Table 1** presents a summary of the current research on the role of pathogens and their byproducts in the induction of pyroptosis in periodontal tissue, providing an update on the comprehensive understanding of the possible effects of pyroptosis on periodontal diseases. As displayed in **Table 1**, studies restricted their focus on caspase-1/GSDMD, the role of caspases, GSDMs, granzymes, and ELANE, which, beyond caspase-1/GSDMD, remain to be fully elucidated in periodontitis. Also, the favorable aspect of pyroptosis in macrophages, which might contribute to the clearance of periodontal pathogens, needs to be clarified.

**Table 1:** Reported periodontal pathogenic factors in the activation of pyroptosis-related caspases and gasdermins.

Tissue and/or cells	Pyroptosis inducers	Inflammasome	Caspases	Gasdermins	Reference
THP-1	*Mycoplasma salivarium*	NLRP3	Caspase-1	N/A	Sugiyama et al., 2016
	*T. denticola* surface protein Td92	NLRP3	Caspase-1	N/A	Jun et al., 2012
	LPS from *E. coli*	NLRP3	Caspase-1/4	GSDMD	Li et al., 2020
	*E. faecalis*	NLRP3	Caspase-1	GSDMD	Ran et al., 2021
U937	*P. gingivalis*	NLRP3	Caspase-1/11	GSDMD	Li et al., 2019
XS106	*Mycoplasma salivarium*	NLRP3	Caspase-1	N/A	Sugiyama et al., 2016
HPDLCs	Cyclic stretch	NLRP1 and NLRP3	Caspase-1/5	GSDMD	Zhao et al., 2016; Zhuang et al., 2019
	*P. gingivalis* and LPS	NLRP3	Caspase-4	GSDMD	Chen et al., 2021
HGEs	Butyrate	N/A	Caspase-3/1/5	GSDME	Liu et al., 2019
HGFs	*T. denticola* surface protein Tp92	N/A	Caspase-4	GSDMD	Jun et al., 2018
	LPS from *E. coli*	NLRP3	Caspase-1	GSDMD	Huang et al., 2020
	Combination of hypoxia and LPS from *P. gingivalis*	NLRP3	Caspase-1	GSDMD	Yang et al., 2021
	*P. gingivalis*	NLRP6	Caspase-1	GSDMD	Liu et al., 2018
	LPS from *P. gingivalis*	NLRP3	Caspase-1/4/5	GSDMD	Li et al., 2021
Human macrophages from blood	*A. actinomycetemcomitans* leukotoxin	N/A	Caspase-1	N/A	Kelk et al., 2011
HSC-2; HOMK107; Immortalized mouse macrophages	Dental calculus	NLRP3	Caspase-1	N/A	Ziauddin et al., 2018
RAW 264.7 co-cultured with HPDLSCs	Hyperglycemia	NLRC4	Caspase-1	GSDMD	Zhao et al., 2021
RAW 264.7; Mice gingival tissue	High glucose, diabetes, and LPS from *P. gingivalis*	AIM2 and NLRP3	Caspase-1	GSDMD	Nie et al., 2021; Zhou et al., 2020
Murine bone-marrow-derived macrophages; Human monocyte-derived macrophages	*P. gingivalis* and its outer membrane vesicles	NLRP3	Caspase-1	N/A	Fleetwood et al., 2017
Oral epithelial cell	*Streptococcus sanguinis*	N/A	Caspase-1/3/7	N/A	White et al., 2020
MG63 cells	LPS from *E. coli*	NLRP3	Caspase-1	GSDMD	Liu et al., 2020
HPDLFs	LPS from *P. gingivalis* and *E. coli*	NLRP3	Caspase-1	N/A	Cheng et al., 2018

XS106, murine epidermal-derived Langerhans cell line; THP-1, human acute monocytic leukemia cell line; HPDLCs, human periodontal ligament stem cells; HSC-2, human oral squamous carcinoma cells; HOMK107, human primary oral epithelial cells; HGFs, human gingival fibroblasts; RAW 264.7, murine macrophage line; MG63, human osteosarcoma MG63 cell line; U937, human myelomonocytic cell line.

Advances in the field have led to a more detailed understanding of how pyroptosis is regulated. Recent studies have shown that Ninjurin-1 mediates the breakdown of the plasma membrane to smaller pieces after pyroptosis induced by GSDMD (Kayagaki et al., 2021), thus amplifying inflammation and helping clear pathogens. The Ragulator-Rag complex of mTORC1 is reported to be necessary for GSDMD pore-forming activity in macrophages (Evavold et al., 2021). Posttranslational modification of GSDMs also contributes to the regulation of pyroptosis. For example, it has been reported that the Shigella ubiquitin ligase IpaH7.8 mediates the ubiquitylation of N-terminal PFD in human GSDMD and GSDMB and promotes their degradation in immune cells to prevent pyroptosis, enabling infection (Hansen et al., 2021; Luchetti et al., 2021). In addition, succination at C192 of the N terminal of GSDMD reduces its binding to caspase-1, thus blocking its processing and oligomerization and preventing pyroptosis-induced cell death (Humphries et al., 2020). Moreover, chemotherapy drugs were reported to promote pyroptosis by palmitoylation of GSDME (Hu et al., 2020). However, these newly discovered regulation mechanisms have not been verified in periodontitis.

Despite accumulating achievements in this field recently, few studies have focused on the basic molecular mechanism of pyroptosis in periodontitis and the potential favorable aspects of pyroptosis in periodontitis, and whether the recently discovered regulation mechanisms also participate in the process of periodontitis are still unknown.

## Role of pyroptosis-regulated cell death mechanisms in periodontitis

Pyroptosis in periodontal tissue has been demonstrated to induce inflammation, resulting in periodontal tissue damage (**Table 1**). Pyroptosis-related inflammasomes, caspases, and cytokines have been proven to be tightly associated with inflammatory diseases and play important roles in pathogen defense, bone resorption, and regeneration of various tissues. Among all the pyroptosis-related inflammasomes and caspases, NLRP3 and caspase-1 are the most comprehensively characterized members in periodontitis. One study found that caspase-1 deficiency suppressed the secretion of inflammation factors from macrophages and alleviated bone resorption in periodontal tissue during periodontitis (Rocha et al., 2020). These authors also found that NLRP3 deficiency did not contribute to this process (Rocha et al., 2020). However, this is inconsistent with the findings of (Zang et al., 2020), which indicated that aged mice lacking NLRP3 showed better bone mass, and inhibition of the activation of NLRP3 with MCC950 significantly suppressed alveolar bone loss (Zang et al., 2020). In line with this result, Chen also found that in mice with ligature-induced periodontitis, NLRP3 deficiency decreased osteoclast precursors and suppressed osteoclast differentiation and alveolar bone destruction (Chen et al., 2021). These contradictory results might be attributed to the different ages of the studied mice; however, this hypothesis requires clarification in future studies.

The activation of inflammasomes and caspases ultimately leads to the maturation and secretion of inflammatory factors; the active forms of IL-1β and IL-18 are the direct byproducts of this process. IL-1β is considered to be the most important factor contributing to periodontal tissue damage. It is expressed in macrophages, dendritic cells, GFs, PDLCs, and osteoblasts. IL-1β was markedly increased in the saliva of patients with periodontitis and gingivitis, as compared to that of healthy individuals (Lira-Junior et al., 2021). Evidence shows that elevated IL-1β levels caused by pyroptosis play a direct role in the pathophysiological process of periodontitis (Cheng et al., 2020). Previous studies have reported that elevated IL-1β enhanced extracellular matrix degradation and bone resorption by directly upregulating collagenolytic enzymes and matrix metalloproteinases (MMPs) in periodontal tissues (Panagakos et al., 1994; Polzer et al., 2010; Schett et al., 2016). IL-1β was also found to upregulate receptor activator for NF-kB ligand (RANKL) expression and promote osteoclast genesis directly, thus promoting inflammatory bone loss (Bloemen et al., 2011; Huynh et al., 2017). In line with this, IL-1β was also proved to promote inflammatory cell infiltration toward alveolar bone in experimental periodontitis (Graves et al., 1998), thus further aggravating alveolar bone loss.

IL-1β certainly activates inflammatory pathways and triggers the release of other inflammatory factors from various cell types, amplifying the inflammatory pathway signals and mediating the amplification of the inflammatory damage caused by pyroptosis. For example, it stimulated chondrocytes to synthesize IL-8, TNF-α, and IL-6 (Guerne et al., 1990; Lotz et al., 1992; Xu et al., 2021), promoted the expression of IL-6 and TNF-α in human retinal microvascular endothelial cells (Giblin et al., 2021), and facilitated the secretion of IL-1a, IL-8, and IL-18 from fibroblast-like synoviocytes (Kim et al., 2021). More importantly, IL-1β was evidenced to promote the secretion of C-C motif chemokine ligand (CCL) 20 and C-X-C motif chemokine ligand (CXCL) 10 from HPDLSCs (Long et al., 2001; Zhu et al., 2012; Hosokawa et al., 2015); IL-2, IL-6, IL-8, IL-23, interferon (IFN)-γ, IL-13, and TNF-α from HPDLFs (Abidi et al., 2020); and prostaglandin E2 (PGE2), IL-6, and IL-8 from HGFs (Ono et al., 2011). These elevated inflammatory factors in periodontal tissue ultimately lead to increased inflammation in periodontitis. However, IL-1β was reported to have dual roles in the osteogenesis of periodontal ligament stem cells (PDLSCs), where low doses of active IL-1β can promote the osteogenesis of PDLSCs through the BMP/Smad pathway, while higher doses of IL-1β can inhibit osteogenesis through the activation of NF-κB and MAPK signaling (Mao et al., 2016). This needs to be verified in pyroptosis-mediated periodontitis, which is usually a severe inflammatory condition. Besides, IL-1β is also reported to upregulate apoptotic signaling pathways (Guadagno et al., 2015; Liang et al., 2018; Wang et al., 2019; Kang et al., 2021), autophagy (Romagnoli et al., 2018), and oxidative stress and is thus involved in tissue damage through inflammatory independent pathways (Zhu et al., 2015; Wang et al., 2021).

IL-18 belongs to the IL-1 family. Its roles in viral, bacterial, parasitic, and fungal infections have been comprehensively studied. Polymorphism of the IL-18 gene was demonstrated to be associated with periodontitis (Shan et al., 2020). In one study, RANKL and periodontal bone loss were proved to be evoked in IL-18 transgenic mice (Yoshinaka et al., 2014). Studies have also reported that IL-18 stimulation promotes proinflammatory cytokine production in periodontal ligament cells, including IFN-γ, IL-2, and TNFα (Vecchie et al., 2021). When infected with P. gingivalis, neutralization of IL-18 inhibited the release of cytokines, chemokines, and MMPs, i.e., IL-1β, IL-6, IL-8, and MMP-1/8/9. This also contributes to decreasing the recruitment of inflammatory cells into periodontal tissues and to less alveolar bone resorption (Zhang et al., 2021). In addition, IL-18 is reported to promote the secretion of matrix metalloproteinases in HPDLFs by activating NF-kB signaling (Wang et al., 2019). Previous studies have indicated that IL-18 blockade is a promising therapeutic target for rheumatic diseases and infantile-onset macrophage activation syndrome (Vecchie et al., 2021). Given its role in regulating the inflammation damage associated with periodontitis, IL-18 blockade may also be an option for the treatment of periodontitis. Further detailed studies are warranted to investigate the exact role of IL-18 in periodontitis.

**Figure 1:**
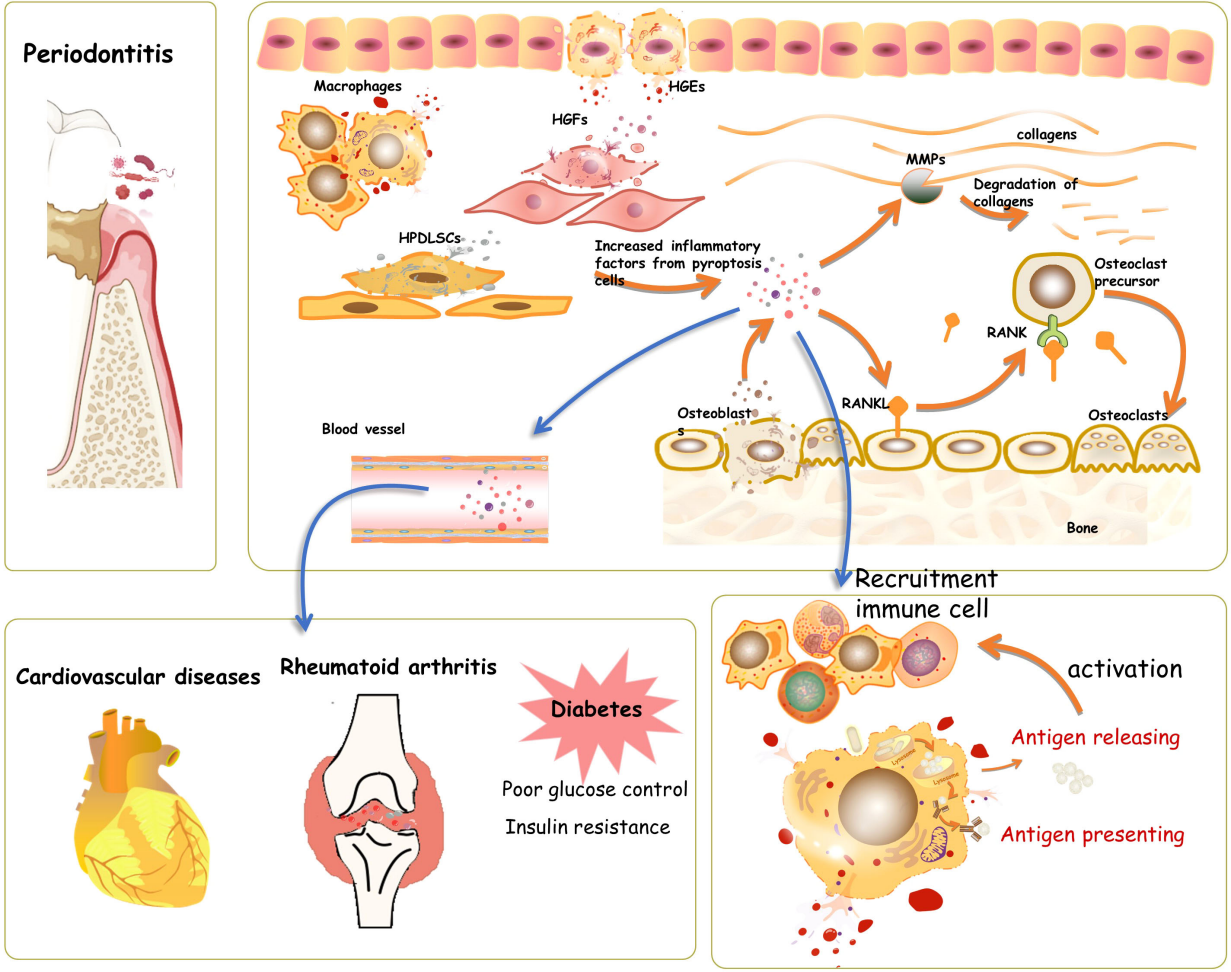
Pyroptosis in periodontal tissue. During the process of periodontitis, pyroptosis takes place in human gingival epithelial cells (HGEs), human gingival fibroblasts (HGFs), osteoblasts, and human periodontal ligament stem cells. The induced pyroptosis involves the activation and recruitment of immune cells, thus helping clear and prevent the spread of pathogens. However, the hyperactive and long-lasting pyroptosis would accelerate the process of periodontitis by increasing cell death and elevating inflammatory factors, which mediate the activation of oxygen species and matrix metalloproteinases, further causing connective tissue damage in periodontal tissue directly. The cascade amplification of the inflammatory response during pyroptosis might also contribute to the aggravation of systematic diseases, such as cardiovascular disease, diabetes, and rheumatoid arthritis.

Taken together, the literature shows that an increased level of pyroptosis in periodontitis can promote the secretion of active inflammatory factors (IL-1β, IL-18), thus amplifying the inflammation response, leading to an overactive immune response; this ultimately decreases bone formation, enhances bone resorption by upregulation of RANKL, exacerbates the destruction of periodontal tissue, and suppresses its regeneration (**Figure 1**). Although persistent localized pyroptosis could enhance periodontal tissue disruption and pathogen dissemination, pyroptosis has also been found to limit pathogen replication, enhance innate and adaptive immune responses, and improve host survival in other tissues (Jorgensen et al., 2016), as a recent study found that GSDMD deficiency diminished neutrophil-killing responses against Escherichia coli infection (Kambara et al., 2018). However, whether this favorable aspect of pyroptosis can help clear pathogens in periodontal tissue has yet to be addressed experimentally.

## Pyroptosis in the relationship between periodontitis and systemic disease

Emerging evidence demonstrates that pyroptosis might be involved in the bidirectional relationship between periodontitis and systemic diseases, such as diabetes, cardiovascular diseases, and rheumatoid arthritis (Jain et al., 2021). These relationships are further summarized below.

### Diabetes

The association between diabetes and periodontitis has been thoroughly investigated (Jain et al., 2021; Pirih et al., 2021). Diabetes is characterized by chronic subclinical inflammation, which is directly involved in the pathogenesis of diabetes-associated periodontitis (Pirih et al., 2021). In addition, diabetes induces increased advanced glycation end-products (AGEs) (Yu et al., 2012; Zizzi et al., 2013; Mei et al., 2019), oxidative stress (Tothova and Celec, 2017; Buranasin et al., 2018), an unbalanced periodontal microbiome (Shi et al., 2020; Sun et al., 2020; Balmasova et al., 2021), and immune dysfunction (Lazenby and Crook, 2010; Zhu and Nikolajczyk, 2014), which also contribute to the progression of periodontitis. Recently, considerable attention has been directed to pyroptosis caused by diabetes and its role in diabetes complications. The induction of pyroptosis in human retinal microvascular endothelial cells through the AGEs/P2X7R/NLRP3/GSDMD pathway was found to contribute to the progression of diabetic retinopathy (Yang et al., 2020). (Chai et al., 2021) reported that intermittent high glucose induces pyroptosis of cardiomyocytes through NLRP3/caspase-1/GSDMD (Chai et al., 2021). Moreover, AGEs produced by high-glucose induced pyroptosis in human kidney-2 cells through AGEs/NLRP3/GSDMD were found to accelerate the kidney damage caused by diabetes (Li et al., 2020). Hyperglycemia also leads to an increase in NLRP3 in diabetic muscle cells, further upregulating the pyroptosis pathway in muscle cells (Aluganti Narasimhulu and Singla, 2021). There is also accumulating evidence indicating that pyroptosis mediates the adverse outcomes of diabetes-associated periodontitis. (Nie et al., 2021) reported that hyperglycemia triggers gingival destruction and impairment of macrophage function, including inflammatory cytokine secretion, phagocytosis, chemotaxis, and immune responses (Nie et al., 2021). In line with this, (Zhao et al., 2021) also reported that hyperglycemia promotes GSDMD-dependent pyroptosis of macrophages through NLRC4 phosphorylation, thus activating NF-κB signaling in HGFs and further aggravating periodontitis (Zhao et al., 2021). Diabetes also inhibits the proliferation and differentiation of osteoblasts in alveolar bone by activating the caspase-1/GSDMD/IL-1β pathway (Yang et al., 2020). Given the evidence suggesting that pyroptosis mediates the progression of diabetes-induced periodontitis, pyroptosis may serve as a new therapeutic target for diabetes-associated periodontitis. However, more comprehensive research on pyroptosis in diabetes-associated periodontitis is warranted to fully understand its mechanism.

Periodontitis also participates in the pathological process of diabetes, as has been reviewed by others (Lalla and Papapanou, 2011; Preshaw et al., 2012; Polak and Shapira, 2018; Pirih et al., 2021). Increased circulating levels of different inflammatory cytokines are associated with poor glucose control and insulin resistance (Polak and Shapira, 2018). IL-1β, a byproduct of pyroptosis, has been suggested to link periodontitis and systemic disorders (Zhu et al., 2015); however, this has not been validated in diabetes-associated periodontitis. Therefore, the exact mechanisms by which periodontitis-induced pyroptosis affects the progression of diabetes are largely unknown. Both in vivo and in vitro studies are needed to address this issue.

### Cardiovascular diseases

Cardiomyocytes, macrophages, vascular smooth muscle cells, endothelial cells, and fibroblasts in the cardiovascular system can undergo pyroptosis, resulting in the progression of cardiovascular diseases (Fidler et al., 2021; Jan et al., 2021; Yao et al., 2021; Zhou et al., 2021). This was also evidenced by alleviated cardiac damage in NLRP3 knockout mice (Busch et al., 2021). Therefore, NLRP3-mediated pyroptosis has been considered a candidate for cardiovascular disease treatment.

Periodontitis has been suggested to be the source of inflammation resulting in cardiovascular diseases. However, patients with periodontitis and cardiovascular diseases often suffer from diabetes and are tobacco users, which can also increase systemic inflammation, making it challenging to prove that periodontitis is the direct source of inflammation resulting in cardiovascular disease. Nonetheless, periodontal pathogenic bacteria, such as P. gingivalis, have been confirmed to be present in atherosclerotic plaques (Haraszthy et al., 2000; Pavlic et al., 2021). The colonized periodontal pathogens in the cardiovascular system were suggested to come from transient bacteremia caused by dental procedures, such as daily tooth care, tooth extraction, scaling, and periodontal probing. This also contributes to periodontal bacteria entering the circulation and inducing low-grade inflammation. Moreover, bacterial metabolic products, such as endotoxins and gingipains, can also trigger systemic inflammatory responses, and some of them have been shown to induce pyroptosis in other tissues (Naderi and Merchant, 2020; Jain et al., 2021). However, whether pyroptosis participates in periodontitis-associated cardiovascular diseases remains largely unknown.

### Rheumatoid arthritis

Studies have reported that periodontitis-related pathogens and their byproducts can disturb the immune balance. Invasive P. gingivalis promotes the occurrence and development of collagen-induced arthritis in mice through the inhibition of the process of B-cell differentiation into B10 cells (Zhou et al., 2021). In addition, P. gingivalis can produce peptidyl-arginine deiminase, thus increasing the formation of anticyclic citrullinated peptide. Moreover, P. gingivalis–induced gingipain mediates the degradation of fibrinogen and alpha-enolase, which provides more substrates for citrullination (Wegner et al., 2010; Quirke et al., 2014). Pyroptosis was reported to participate in elevated inflammation in rheumatoid arthritis (Li et al., 2019; Wu et al., 2020). Thus, we speculate that pyroptosis might be involved in the pathophysiology of periodontitis-associated rheumatoid arthritis. However, this requires further clarification.

## Crosstalk between pyroptosis and other forms of programmed cell death

### Pyroptosis and apoptosis

Pyroptosis was traditionally conceived to have a distinct morphology and undergo distinct pathways from apoptosis; nonetheless, this has been challenged with more recent evidence showing connections between pyroptosis and apoptosis. Apoptosis was the first characterized form of programmed cell death and can be classified as either extrinsic or intrinsic apoptosis. Caspase-3 has traditionally been regarded as a critical effector of apoptosis-induced cell death in intrinsic apoptosis. Recent advances have indicated that caspase-3/GSDME might be a switch between apoptosis and pyroptosis (Jiang et al., 2020). A study by (Jiang et al., 2020) reported that in coral, GSDME is cleaved by caspase-3 at two tetrapeptide motifs, 238DATD241 and 254DEPD257, yielding two active isoforms of the N-terminal domain of GSDME that are capable of inducing pyroptosis (Jiang et al., 2020). (Rogers et al., 2017) reported that during apoptosis in 293T cells, caspase-3 cleaves the 267DMPD270 domain of GSDME, which targets the plasma membrane to induce pyroptosis (Rogers et al., 2017). In peri-implantitis and periodontitis, this signal pathway has been found to mediate TNF-α- and butyrate-triggered pyroptosis in human gingival epithelial cells, respectively (Liu et al., 2019; Chen et al., 2021). The relationship between pyroptosis and apoptosis was also verified by the finding that exposure to cadmium, an apoptosis trigger, can evoke the caspase-1 mediated pyroptosis pathway (Wei et al., 2020). Caspase-8, the initiator of extrinsic apoptosis, has also been found to be involved in the pyroptosis signaling pathway (Sarhan et al., 2018; Fritsch et al., 2019; Zhang et al., 2021). (Sarhan et al., 2018) reported that caspase-8 mediated pyroptosis during Yersinia infection in macrophages by directly binding to GSDMD, driving the cleavage of GSDMD (Orning et al., 2018; Sarhan et al., 2018). In addition, caspase-8 has been found to mediate the cleavage of GSDMC after α-ketoglutarate treatment in Hela cells, thus promoting pyroptosis (Zhang et al., 2021). Furthermore, caspase-8 was found to stimulate caspase-3-dependent GSDME cleavage, further facilitating pyroptosis (Li et al., 2021). These results indicate that caspase-8 is another molecular switch beyond caspase-3 that controls apoptosis and pyroptosis (Fritsch et al., 2019). The roles of granzyme A and B in apoptosis have been thoroughly studied (Martinvalet et al., 2005; Velotti et al., 2020). Recently, it has been discovered that they are also able to directly cleave GSDMs, as discussed above, further confirming the connection between apoptosis and pyroptosis.

In turn, the upstream signals of pyroptosis have also been reported to evoke apoptosis pathways. Oligomerization of the initiating proteins, such as AIM2 and NLRP3, was found to promote apoptosis through the recruitment of caspase-8 by ASC, thereby initiating apoptosis (Sagulenko et al., 2013). During pyroptosis, activated caspase-1 can bidirectionally lead to the activation of caspase-3/8/9-related apoptosis (Tsuchiya et al., 2019). More recently, the N-terminal of GSDME was found to permeabilize the mitochondrial membrane, releasing cytochrome c and thus activating apoptosis through the intrinsic apoptosis pathway (Yang et al., 2020). However, the role of pyroptosis-dependent secondary apoptosis in periodontitis is unclear.

**Figure 2:**
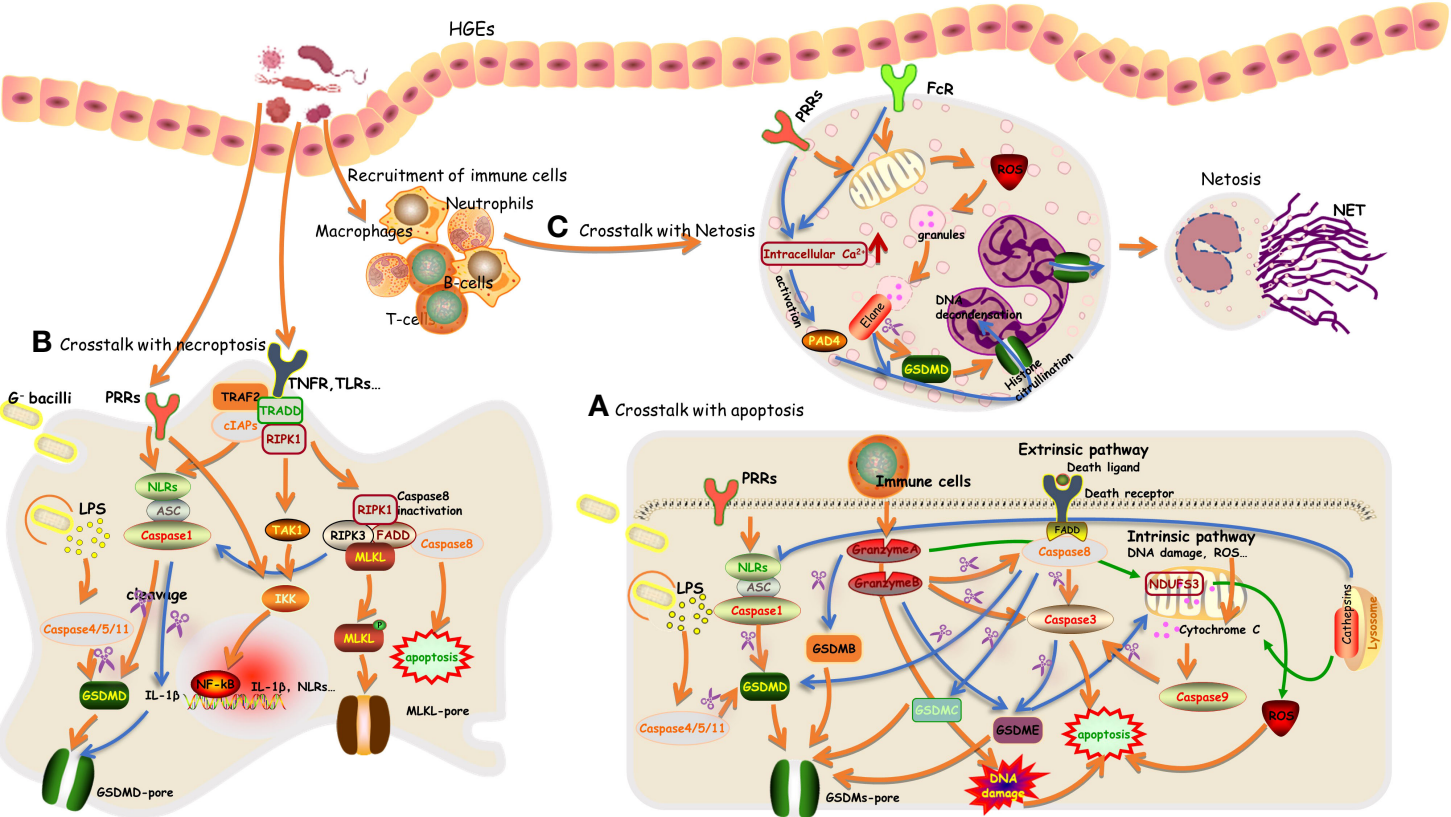
An overview of the crosstalk between pyroptosis with apoptosis, necroptosis, and NETosis in periodontal tissue **(A–C)**.

In sum, these results confirm the presence of a bidirectional relationship between pyroptosis and apoptosis and further suggest that pyroptosis tends to be concurrent with apoptosis (Xi et al., 2016; Doitsh et al., 2017) (**Figure 2**). However, cells or tissues are usually dominated by one programmed death pathway in response to a certain trigger, and this can be attributed to the expression levels of GSDMs and certain caspases. Deficiency of pyroptosis-associated caspases or GSDMs switches pyroptosis to apoptosis; for example, deficiency of GSDMD reverts the cell-death morphology to apoptosis upon the activation of caspase-1 or caspase-8 (Pierini et al., 2012; Sarhan et al., 2018; Tsuchiya et al., 2019; Jiang et al., 2020; Zhang et al., 2020). A sufficient amount of substrates for pyroptosis contributes to the initiation of pyroptosis (Wang and Kanneganti, 2021). In addition, the cell type has also been suggested to influence the undergoing form of programmed death (Yang et al., 2020). This requires further clarification in various periodontal cell types.

Caspase-1/4/5/11/3/8 levels have all been reported to be increased in periodontitis (Hirasawa and Kurita-Ochiai, 2018; Jun et al., 2018; Liu et al., 2018). Elevated expression and activity of caspase-3 and caspase-8 certainly participate in aggravating the progression of periodontitis through the apoptosis pathway (Alikhani et al., 2004; Hirasawa and Kurita-Ochiai, 2018; Aral et al., 2019). However, no studies analyzed apoptosis–pyroptosis interactions during this process, and whether pyroptosis can be activated simultaneously and participate in the progression of periodontitis is unclear. Given the accumulating evidence of a connection between apoptosis and pyroptosis, we speculate that the interaction between the two might contribute to the pathophysiology of periodontitis. The role of the bidirectional relationship between pyroptosis and apoptosis in periodontitis needs to be investigated in more detailed mechanistic studies.

### Pyroptosis and necroptosis

Necroptosis is another well-defined form of programmed cell death. It is initiated by the activation of tumor necrosis factor receptor (TNFR), death receptor, TLR, or type I interferon receptor (IFNAR). This is followed by the formation of complex I on the membrane, which contains TNFR-associated death domain (TRADD), Fas-associated death domain (FADD), TNFR-associated factors (TRAFs), receptor-interacting protein 1(RIPK1), and cellular inhibitor of apoptosis protein 1 and 2. Then, there is the recruitment of RIPK3 and the formation of the RIP complex, leading to the phosphorylation and activation of mixed lineage kinase domain-like (MLKL). Activated MLKL mediates pore formation, which facilitates the release of inflammatory factors and cellular components, ultimately resulting in the collapse of the membrane (Frank and Vince, 2019; Yuan et al., 2019; Bertheloot et al., 2021). This process has already been summarized in detail. As alluded to above, necroptosis occurs through a discriminate pathway compared with pyroptosis. In addition, there are also distinct morphologies: cells that undergo necroptosis usually exhibit loose cellular detachment and exaggerated cellular swelling due to the ion-selective MLKL pores (Frank and Vince, 2019). Nonetheless, they still interact with each other, as the previously defined receptors for initiating necroptosis have also been demonstrated to be involved in pyroptosis pathways (Frank and Vince, 2019). Triggers such as P. gingivalis and LPS have been identified to induce both pyroptosis and necroptosis in periodontitis (Ke et al., 2016; Geng et al., 2022; Yang et al., 2022). More recently, RIPK1, RIPK3, and MLKL have been identified to be involved in the mediation of the activation of NLRP3/caspase 1, which might contribute to the activation of pyroptosis during periodontitis (Conos et al., 2017; Speir and Lawlor, 2021). These results suggest the simultaneous existence of these two forms of death in periodontal tissue. Unlike apoptosis, which is immunologically silent, the excessive activation of pyroptosis and necroptosis consequently elicits a destructive immune response that participates in the process of periodontitis. The complex interaction of these pathways in periodontitis is largely unknown, and the primary inflammatory programmed death pathway in distinct periodontal cells has yet to be deciphered. Understanding this topic will provide us with new opportunities for potential clinical treatments of periodontitis.

### Pyroptosis and NETosis

There is growing evidence indicating neutrophils as crucial regulators in periodontitis. In response to pathogens, the recruited neutrophils exert their host defense effects by releasing cytotoxic factors and enzymes, phagocytosing pathogens, and discharging neutrophil extracellular traps (NETs) during NETosis (Hajishengallis, 2020). NETosis is initiated by the activation of neutrophils and, simultaneously, changes in intracellular calcium concentration, the elevation of reactive oxygen species, and the activation of kinase signaling cascades contributing to the formation of NETs (Thiam et al., 2020). NETs are intercellular chromatins decorated with activated protease (such as elastase and myeloperoxidase), acting as scaffolds to trap and obliterate pathogens. The release of intracellular contents, including DNA, histones, and proteins, during NETosis, contributes to the autoimmune response, which directs research on NETosis mainly to the field of autoimmune diseases. Limited evidence in periodontitis showed that periodontal pathogens F. nucleatum, Aggregatibacter actinomycetemcomitans, and P. gingivalis contribute to the activation of NETosis (Hirschfeld et al., 2016; Alyami et al., 2019; Bryzek et al., 2019). P. gingivalis–induced NETosis was gingipains dependent, and gingipains in turn mediated the proteolysis of components of NETs (Bryzek et al., 2019). This might lead to the amplification of the accumulation of NETs during periodontitis and further aggravate tissue damage caused by neutrophils. These NETosis-invoked pathogens have also been reported to trigger pyroptosis in other types of cells in periodontal tissues. The key factors that influence neutrophils to undergo NETosis, pyroptosis, or phagocytose when responding to identical triggers and pathogens remain unclear. In essence, whether neutrophils can undergo pyroptosis is still controversial (Sollberger, 2022). The large amount of neutrophil proteases and the low expression level of caspases and inflammasomes determine the priority for NETosis in neutrophils. In addition, this was also evidenced by neutrophil protease-mediated activation of GSDMD, and activated inflammasomes and GSDMD tend to participate in the release of chromatin in a feed-forward loop to promote NETosis instead of inducing pyroptosis (Chen et al., 2018; Sollberger et al., 2018). Researchers also failed to detect pyroptosis morphologies during this process. This has been summarized in **Figure 2**. However, these were not explored in periodontitis.

### Pyroptosis, apoptosis, and necroptosis are interconnected

The recent progress in the research on cell death advanced our understanding of the extensive and intricate crosstalk between different cell death signaling cascades. Caspase-8 was proposed as a master regulator of apoptosis, necroptosis, and pyroptosis. Signal transduction via LPS binding to TLRs results in the activation of caspase-8, RIPK1, and RIPK3, thus contributing to the activation of necroptosis. During this process, mammalian inhibitor of apoptosis (IAP) proteins, including X-linked IAP (XIAP), cellular IAP1, and IAP2 (cIAP1/2), can bind caspase-8, caspase-3, and caspase-7 directly and inhibit their apoptotic caspase activity; this depends on the BIR domains in IAPs (Silke and Meier, 2013; Bertheloot et al., 2021). The combined loss of XIAP and cIAP1/2 enhanced the caspase-8-mediated apoptotic pathway and cleavage of pro-IL-1β. In parallel, RIPK3-mediated necroptosis and activation of NLRP3/IL-1β were also enhanced (Tenev et al., 2011; Moulin et al., 2012; Lawlor et al., 2015). Furthermore, tumor necrosis factor-related apoptosis-inducing ligand decoy receptors, survivin and XIAP, have been suggested to suppress the apoptosis of inflammatory cells in periodontitis and are thus associated with a prolonged lifespan of inflammatory cells (Lucas et al., 2010). Caspase-8 deficiency failed to suppress the maturation of IL-1β but enhanced the recruitment of RIPK1, followed by phosphorylation of RIPK3, and ultimately contributed to MLKL-mediated necroptosis and the activation of NLRP3/IL-1β in pyroptosis (Fritsch et al., 2019). Notably, RIPK1 is required to limit RIPK3 and caspase-8 mediated cell death; this was evidenced by mice with Ripk1 deficiency dying soon after birth due to uncontrolled cell death depending on caspase-8, RIPK3 (Rickard et al., 2014; Samir et al., 2020), or RIPK3 mediated activation of NLRP3/IL-1β. However, whether activated NLRP3/IL-1β was involved in the activation of pyroptosis was not clear. Presumably, this depended on the cellular substrate content and the specific type of cell and tissue. c-FLIP is also identified as a checkpoint to control the activation of caspase-8. c-FLIP was upregulated in LPS-primed macrophages. The elevated short isoform of c-FILP inhibited caspase-8-dependent apoptosis by disrupting pro-caspase-8 oligomer assembly (Speir and Lawlor, 2021). Correspondingly, NLRP3/IL-1β and necroptosis signaling were evoked. Contradictorily, the caspase-8-cFLIP (long isoform) complex is required for the inhibition of both apoptosis and necroptosis to suppress cell death (Van Opdenbosch et al., 2017). These results indicate the intricate connection between cell death pathways and imply that different periodontal cell types could undergo distinct cell death pathways during periodontitis. However, these need to be clarified in future explorations.

PANoptosis, another newly recognized proinflammatory programmed cell death pathway, is executed by the PANoptosome, which contains molecules involved in the pyroptotic, apoptotic, and necroptotic pathways. This new definition of cell death highlights the crosstalk and concurrence of these three pathways. The PANoptosome was regarded as a platform that acts as a sensor and executor during infection; it was initially shown to consist of NLRP3, NLRC4, AIM2, ASC, TNFR1, RIPK1, RIPK3, MLKL, caspase-1/3/7/8, and GSDMD (Samir et al., 2020). Recently, ZBP1 was recognized as an apical sensor by recruiting RIPK3 and caspase-8 during IAV infection. This was evidenced by ZBP1 deficiency completely abolishing IAV-induced PANoptosis (Zheng and Kanneganti, 2020). Caspase-6 was proved to facilitate the interactions between RIPK3 and ZBP1 in this process (Zheng et al., 2020). These results extend our understanding of the participating molecules. However, Sendai virus and respiratory syncytial virus were demonstrated to trigger PANoptosis through ZBP1-independent pathways, implying different PANoptosome components in response to specific microbes (Place et al., 2021). Hitherto, no study has focused on this topic with regard to periodontitis. Considering the critical role of PANoptosis in defending and restricting pathogens, exploring it in periodontal disease might be helpful in the development of new treatment strategies in this field.

## Pyroptosis: A new therapeutic opportunity for periodontitis

Appropriate pyroptosis plays a key role in the clearance of pathogens by removing intracellular replication niches and enhancing the host’s defense responses (Bergsbaken et al., 2009; Jorgensen et al., 2016; Hansen et al., 2021). The adequate release of cytokines is critical for immune activity, contributing to tissue angiogenesis and repair. However, overactivation of pyroptosis can result in a massive inflammatory response, leading to aggravation of damage to tissues and organs; moreover, long-term exposure to an inflammatory environment can also increase the risk of diabetes, cancers, and so on (Schroder et al., 2010; Hou et al., 2021). Studies have reported elevated levels of pyroptosis in periodontitis, and pharmacological inhibition of pyroptosis prevents the progression of periodontitis. For example, the caspase-1 inhibitor vx-765 was found to suppress bone loss and inhibit the expression of inflammatory factors (IL-1β, MCP-1, IL-6, and IL-8) in an experimental AP rat model (Cheng et al., 2018). Another caspase-1 inhibitor, Z-YVAD-FMK, was found to suppress gingivitis caused by LPS (Li et al., 2021). In addition, the NLRP3 inhibitor MCC950 restored the osteogenic function of MG63 cells under LPS stimulation (Liu et al., 2020). Also, the pan-caspase inhibitor Z-VAD-FMK and the caspase-4 inhibitor Z-LEVD-FMK have been found to alleviate inflammation in PDLSCs exposed to P. gingivalis (Chen et al., 2021). Knockdown of GSDMD has been found to alleviate P. gingivalis–related inflammation in HGFs (Zhao et al., 2021). These results showed that pharmacological inhibition of canonical or noncanonical pyroptosis pathways or gene knockout of pyroptosis-associated molecules results in significantly reduced alveolar bone loss and periodontal tissue inflammation and damage. Given the elevated pyroptosis level in periodontal tissue during periodontitis and its role in the pathological progression of periodontitis, treatments targeting pyroptosis will provide another effective way to cope with periodontitis. However, at present, the available inhibitors of pyroptosis generally act on caspases and inflammasomes, which also participate in other forms of programmed death; to date, there is still a lack of direct and specific pyroptosis inhibitors. Moreover, the role of pyroptosis in periodontitis is only just beginning to be understood; further studies of the signaling pathways involved in pyroptosis should be performed to provide new directions for the treatment of periodontitis.

## Summary

The last decade has witnessed tremendous progress in pyroptosis research with an improved understanding of the role of pyroptosis in inflammatory diseases, cancers, and metabolic and autoimmune diseases. This review has provided a brief overview of the mechanisms of pyroptosis in periodontitis and the most recent research on the mechanisms of pyroptosis in relation to apoptosis. A large number of studies have revealed the critical role of pyroptosis-mediated activation of inflammation in periodontitis; however, as discussed above, the existing studies typically only explored the most characterized pyroptosis pathways. The physiological roles of GSDMs other than GSDMD in periodontitis remain poorly understood. Moreover, knowledge of posttranslational modification and molecular interactions between GSDMs in periodontitis remains incomplete. Therefore, it is critical to continue performing detailed investigations to gain extensive knowledge of pyroptosis in periodontitis. In light of the clear evidence that periodontitis causes systemic inflammation and increases the risk of systemic chronic comorbidities, it can be speculated that pyroptosis may link periodontitis with systemic disease. In this regard, future studies should address the mechanisms of pyroptosis in the connection between periodontitis and systemic diseases. This will facilitate the establishment of pyroptosis-targeted adjunctive treatments, thus contributing to reducing systemic inflammation and promoting systemic health. Crosstalk between pyroptosis and apoptosis, NETosis, and necroptosis has been investigated in various cells and tissues. Caspase-3/8 are considered the switch between apoptosis and pyroptosis, and deficiency in pyroptotic substrates contributes to the switch from apoptosis to pyroptosis. Notably, it seems that pyroptosis is usually concurrent with apoptosis, NETosis, and necroptosis in periodontal tissues during periodontitis. However, these conclusions have not been verified, and it remains unclear which is the primary type of programmed cell death in periodontal tissues during periodontitis. Understanding this topic will help us develop more precise therapeutic methods.

With respect to infectious disease, pyroptosis participates in the clearance of pathogens and enhances the host’s defense responses. On the other hand, long-lasting pyroptosis leads to inflammatory damage in periodontal tissue. While many studies have reported that elevated pyroptosis contributes to the pathophysiological progression of periodontitis, there is limited research exploring the favorable aspect of pyroptosis in the clearance of periodontal pathogens. This proves a challenge for the application of pyroptosis inhibitors in periodontitis. The exploration of the molecules that directly target pyroptosis and the design of experiments to help understand the dual role of pyroptosis in periodontitis will shed light on novel therapeutic opportunities. Overall, it is crucial that pyroptosis be further explored in detail, with the expectation that basic research will translate to clinical practice prevention strategies.

## Author contributions

XHX conceived the structure of the manuscript and wrote the manuscript. XYX, DA, YY, SY, MZ, and HQ collected the materials. TZ and JS reviewed and edited the manuscript. All authors read and approved the final manuscript.

## Funding

This work was financially supported by funding from the National Natural Science Foundation of China (Grant No. 82101014; 81771082; 31971282), China postdoctoral Science Foundation (Grant No. 2021M700628), Natural Science Foundation of Chongqing (Grant No. cstc2021jcyj-bsh0005), and the Chongqing Graduate Tutor Team (2019) (Grant No. dstd201903).

## Conflict of interest

The authors declare that the research was conducted in the absence of any commercial or financial relationships that could be construed as a potential conflict of interest.

## Publisher’s note

All claims expressed in this article are solely those of the authors and do not necessarily represent those of their affiliated organizations, or those of the publisher, the editors and the reviewers. Any product that may be evaluated in this article, or claim that may be made by its manufacturer, is not guaranteed or endorsed by the publisher.

## Abbreviations

GSDMs: Gasdermins; NLRP3: NLR family pyrin domain containing 3; PAMPs: pathogen-associated molecular patterns; AP: Apical periodontitis; IL: interleukin; DAMPs: damage-associated molecular patterns; MHC: major histocompatibility complex; NLRs: NOD-like receptors; ALRs: Absent in melanoma 2 (AIM2)-like receptors; ASC: Apoptosis-associated speck-like proteins containing a caspase recruitment domain; LRR: leucine-rich repeat domain; NBD: nucleotide-binding domain; NACHT: nucleotide-binding and oligomerization domain; PYD: pyrin domain; CARD: caspase recruitment domain; GBPs: guanylate binding proteins; DFNB59: Autosomal recessive deafness-59; ELANE: neutrophil-specific serine protease-neutrophil elastase; HGFs: human gingival fibroblasts; HPDLFs: human periodontal ligament fibroblasts; HPDLSCs: human periodontal ligament stem cells; HGEs: human gingival epithelium; MMPs: matrix metalloproteinases; CCL: C-C motif chemokine ligand; CXCL: C-X-C motif chemokine ligand; IFN: interferon; PGE2: prostaglandin E2; AGEs: Advanced glycation end-products.

## Glossary

**Table:**

GSDMs	Gasdermins
NLRP3	NLR family pyrin domain containing 3
PAMPs	pathogen-associated molecular patterns
AP	apical periodontitis
IL	interleukin
DAMPs	damage-associated molecular patterns
MHC	major histocompatibility complex
NLRs	NOD-like receptors
ALRs	absent in melanoma 2 (AIM2)-like receptors
ASC	apoptosis-associated speck-like proteins containing a caspase recruitment domain
LRR	leucine-rich repeat domain
NBD	nucleotide-binding domain
NACHT	nucleotide-binding and oligomerization domain
PYD	pyrin domain
CARD	caspase recruitment domain
GBPs	guanylate binding proteins
DFNB59	autosomal recessive deafness-59
ELANE	neutrophil-specific serine protease-neutrophil elastase
HGFs	human gingival fibroblasts
HPDLFs	human periodontal ligament fibroblasts
HPDLSCs	human periodontal ligament stem cells
HGEs	human gingival epithelium
MMPs	matrix metalloproteinases
CCL	C-C motif chemokine ligand
CXCL	C-X-C motif chemokine ligand
IFN	interferon
PGE2	prostaglandin E2
AGEs	advanced glycation end-products.
